# Integrating Advanced Practice Nurses in Anesthesia to Tackle Gaps in Current Health Care: A Qualitative Study

**DOI:** 10.1111/nhs.70317

**Published:** 2026-03-08

**Authors:** Luzia Vetter, Balthasar L. Hug, Maya Zumstein‐Shaha

**Affiliations:** ^1^ Department of Nursing Lucerne Cantonal Hospital Lucerne Switzerland; ^2^ Faculty of Health Sciences and Medicine University of Lucerne Luzern Switzerland; ^3^ Department of Medicine Lucerne Cantonal Hospital Lucerne Switzerland; ^4^ Department of Health Bern University of Applied Sciences Bern Switzerland; ^5^ Faculty of Health, Department of Nursing Science University Witten/Herdecke Witten Germany; ^6^ College of Nursing University of Colorado Anschutz Medical Center Aurora Colorado USA

**Keywords:** advanced practice nursing, nurse anesthetists, pain management, perioperative care, quality of health care, recovery room

## Abstract

Aging population and growing shortage of specialists present increasing challenges to anesthetic care. In Switzerland, advanced practice nurses in anesthesia are not yet integrated, despite their potential to contribute to innovative care models. This qualitative study explored current gaps in anesthetic care and assessed the potential role of advanced practice nursing in anesthesia. Semi‐structured interviews were held with 46 participants, including patients, nurse anesthetists, and anesthesiologists. Data were analyzed using Mayring's summarizing content analysis. Patients emphasized “Information and Preparation Needs,” “pain,” and “recurrent hospital stays.” Nurse anesthetists highlighted gaps in “making nursing care visible,” “(interprofessional) practice development,” and “pain therapy.” Anesthesiologists identified challenges in “changes in anesthesia,” the “treatment process,” and “pain management.” The results reveal relevant deficits in perioperative care, underscoring the need for sustainable solutions. Integrating advanced practice nurses into anesthesia could address these gaps by providing patient‐centered preoperative support, fostering interprofessional practice development in the operating room, and providing clinical leadership in the postoperative recovery room and pain management. Advanced practice nursing in anesthesia is a promising strategy to sustainably improve the quality of perioperative health care.

## Key Points

- Contextual factors have led to care gaps in anesthesia, including a lack of advanced practice nursing in the postanesthesia care unit, as well as insufficient support for prehabilitation, patient‐centered care for individuals with chronic conditions, and pain management.
- While nurse anesthetists are already established as specialist nurses in anesthesia, advanced practice nurses (APNs) have not yet been integrated into this field within the Swiss healthcare system, despite their potential to address existing care and leadership gaps.
- Integrating APNs into anesthetic care could foster person‐centered approaches for chronically ill patients and strengthen the quality and continuity of pain management.

## 1 Introduction

Every year, an estimated 313 million surgical procedures are performed under anesthesia worldwide (McDougall and Enright 2018), highlighting its essential role in modern healthcare. Healthcare systems and anesthetic care face severe strain from aging populations, multimorbidity, and staff shortages (Jones and Dolsten 2024). Advances in surgical techniques, such as minimally invasive and robot‐assisted procedures, require highly qualified anesthesia personnel (Hafiani et al. 2024). Rising obesity rates and the opioid epidemic further complicate perioperative care and increase demands on anesthesia teams (Hardt and Wappler 2023; Henshaw et al. 2022). Additionally, fragmented care by interdisciplinary teams often leads to communication issues and treatment delays due to shifting responsibilities (Rosenbaum 2019).

A key structural change is the shift toward outpatient care. In Switzerland, 1.5 million hospital stays in 2023, half for surgery, were contrasted by 4.7 million outpatient hospital cases (Federal Office for Statistics 2024). Outpatient surgery demands precise patient selection, thorough preparation, and evidence‐based discharge criteria. This transition (Trezzinia and Bachb 2020) requires comprehensive perioperative management, including surgical planning, postoperative care, and modern pain strategies, especially given rising opioid dependence (Henshaw et al. 2022). Prehabilitation programs now begin with detailed preoperative assessments of functional, cognitive, emotional, and nutritional status, as well as social support (Bettelli 2023). Consequently, anesthesia now encompasses perioperative medicine, coordinating multidisciplinary teams from planning to full recovery (Royal College of Anesthetists website, cited in Wall et al. 2022).

Traditional patient care no longer meets current demands; interdisciplinary and interprofessional strategies are needed (Grocott et al. 2019). Collaborative coordinated teams embedded in a culture of safety optimize patient outcomes. Joint decision‐making in flat hierarchies allows all team members to contribute their expertise (Nilsson et al. 2020). These models close surgical care gaps, ensure quality, and improve outcomes. Key strategies include coordinated patient pathways, risk prediction, comprehensive data collection, and workforce optimization (Wagstaff and Shenouda 2023).

Implementing collaborative models is challenging. Studies show persistent role ambiguity, limited scopes of practice, and hierarchical tensions between physicians and nurses (Wising et al. 2024). Nurse anesthetists face high task intensity, up to 98 tasks per hour during induction, along with frequent interruptions that affect safety (Olin et al. 2022). High workloads contribute to burnout; in one Dutch survey, 52% of nurses were at high risk of burnout, and 65% reported work‐related errors (Meeusen et al. 2010). Such factors undermine collaboration and workforce optimization.

To achieve healthcare's fourfold goal—better patient experience, population health, reduced costs, and provider well‐being (Sikka et al. 2015)—new care models are needed. Advanced practice nurses (APNs), with a Master of Science in Nursing and evidence‐based decision‐making skills (Schober et al. 2020), can fill critical gaps. APNs improve symptom control, physical functioning, and blood pressure in patients with chronic conditions, and contribute to higher satisfaction, shorter wait times, and lower costs (Htay and Whitehead 2021). They play key roles in collaboration, counseling, leadership, quality improvement, and guideline development, applying research in practice. APNs can specialize in care for specific groups, such as people with dementia or autism in anesthesia (Vetter et al. 2024). While APNs are globally recognized, they are not yet formally integrated into anesthetic care. The role of nurse anesthetists, who have postgraduate specialization, varies worldwide: from a specialist nurse (WHO 2020) to an advanced practice nurse (Meeusen et al. 2010; Stewart et al. 2021). Some models include anesthesia APNs in palliative care teams to leverage their specialized expertise in analgesia, providing independent pain management at home (Vetter et al. 2024).

Despite international recognition of APNs' potential, their integration into anesthesia is contested. Medical associations stress physician oversight due to intraoperative complexity (Hoyem et al. 2019), while nursing organizations advocate for full‐scope APN roles to improve access and reduce costs (Vitale and Lyons 2021). Despite international recognition of APNs' potential, their integration into anesthesia remains contested. These tensions stem from differing views on evidence, professional identities, economic interests, and power dynamics (Hoyem et al. 2019; Vitale and Lyons 2021; Wising et al. 2024). Specifically, conflicting interpretations of evidence regarding patient safety and clinical outcomes influence these professional debates (Dulisse and Cromwell 2010; Sun et al. 2016). Furthermore, professional identities and power dynamics are shaped by persistent hierarchies in the operating room, which can hinder seamless interprofessional collaboration (Wising et al. 2024). These international discourses are particularly relevant for the Swiss context, where formal APN roles in anesthesia have not yet been established.

Large studies show nurse anesthetists operating independently do not increase complications (Sun et al. 2016; Dulisse and Cromwell 2010), though critics note methodological concerns (Sun et al. 2016). Nurses report persistent hierarchies that hinder collaboration (Wising et al. 2024).

In Switzerland, nurse anesthetists receive postgraduate training and play a key role in anesthesia (OdaSanté 2022; SSAPM 2025), but are classified as specialist nurses rather than APNs as defined by WHO (2020). Their education (Diploma of Advanced Studies, NDS HF) consists of 2 years of part‐time study (about 900 theory hours, 30–40 ECTS) plus mandatory clinical training. In contrast, formal APN roles in anesthesia require a master's degree (minimum 90 ECTS), an expanded scope of practice, and autonomous decision‐making. This distinction between specialist nurse anesthetists and APNs is central to our study, which explores whether integrating APNs could address care gaps.

With growing demands on healthcare, evaluating APNs' potential to strengthen anesthetic care is vital. Most international studies examine APN integration retrospectively and from single perspectives (Hoyem et al. 2019; Sun et al. 2016). The Swiss context is underexplored, with few studies capturing multi‐stakeholder views before APN implementation. This study, therefore, explores perceived care gaps in anesthesia in Switzerland from the perspectives of patients, nurse anesthetists, and anesthesiologists, and examines how APNs could support sustainable, future‐oriented anesthetic care.

While nurse anesthetists in Switzerland play a central role, they are currently classified as specialist nurses rather than advanced practice nurses. These international debates are relevant for Switzerland, where formal APN roles in anesthesia are absent and interprofessional dynamics remain largely unexplored. Our study addresses this gap by examining perceived care needs from multiple stakeholder perspectives, providing baseline insights to inform policy and anticipate barriers and facilitators before APN roles are introduced.

## 2 Materials and Methods

A qualitative descriptive design was used to explore perceived needs and the potential role of APNs in anesthesia in Switzerland (Sandelowski 2000). This approach is particularly appropriate when the goal is to provide a comprehensive summary of stakeholder perspectives in everyday language, staying close to the data without imposing a specific theoretical framework (Sandelowski 2010).

Semi‐structured interviews were conducted with patients, nurse anesthetists, and anesthesiologists to collect in‐depth, experience‐based data and to capture diverse viewpoints on current practice gaps and the potential for APN integration. The interview guides are provided in Supporting Information S1. These documents outline the primary questions used to steer the semi‐structured discussions. This study followed the Consolidated Criteria for Reporting Qualitative Research (COREQ) checklist (Tong et al. 2007).

### 2.1 Research Assumptions

The study assumed that patients, nurse anesthetists, and anesthesiologists hold distinct perspectives on care gaps shaped by their specific roles, professional socialization, and hierarchical positions. We anticipated that nurses would emphasize holistic care and communication, which differ from physicians' predominantly biomedical and technical focus. At the same time, patients would contribute unique experiential knowledge, which professionals often overlook. These assumptions justified a multi‐stakeholder design with separate data collection and initial analysis to capture divergent viewpoints and ensure reflexivity.

### 2.2 Sampling and Recruitment

This study was conducted in Switzerland with experts from the German‐speaking part of Switzerland. Patients were recruited from one tertiary hospital serving a catchment area of approximately 800 000 people with a capacity of roughly 700 beds. The hospital offers the full spectrum of surgical services across all disciplines. Participants were recruited from the preoperative anesthesia clinic prior to scheduled elective surgery.

Regarding recruitment, patients undergoing elective surgery were approached consecutively after their anesthesia consultation, with follow‐up interviews conducted postoperatively at home. Emergency cases and individuals with language barriers were excluded, and none had participated in a prehabilitation program. In contrast, anesthesiologists and nurse anesthetists were selected based on their experience in anesthetic practice. Eligible professionals had at least 5 years of experience after graduation as a nurse anesthetist or anesthesiologist. All professional participants were employed at Swiss public hospitals and recruited through their respective professional associations: the Swiss Society for Anaesthesiology and Perioperative Medicine (SSAPM) and the Swiss Association of Nurse Anesthetists (SIGA‐FSIA).

### 2.3 Data Collection

Semi‐structured interview guides were developed based on a literature review and expert consultation. Separate guides were created for patients and professionals, focusing on experiences with anesthetic care, perceived gaps, and views on potential APN integration. Postoperative patient interviews were conducted by telephone at home. Professional interviews were conducted at participants' workplaces or by telephone/video call, at participants' preference. All interviews were audio‐recorded and transcribed verbatim. Interviews lasted between 4 and 28 min. Field notes were taken during and after interviews to capture contextual information and initial impressions.

### 2.4 Researcher Characteristics and Reflexivity

L.V., an experienced qualitative researcher with a background in nursing science, conducted all semi‐structured interviews. L.V. had no prior professional relationships with participants. The interviewer introduced herself as a researcher interested in understanding stakeholder perspectives on anesthetic care and potential APN integration, without disclosing personal views on the topic.

M.Z.‐S. and L.V. conducted data analysis. Both researchers independently coded the transcripts and met regularly to discuss coding discrepancies, refine categories, and ensure analytical rigor. The research team included members with diverse professional backgrounds in nursing and medicine, which facilitated multiple perspectives on the data and enhanced reflexivity throughout the analytical process.

### 2.5 Data Analysis

Qualitative content analysis following Mayring's approach (Mayring and Fenzl 2019) was used to analyze the transcripts. The analysis involved several steps: (1) determining coding units, (2) paraphrasing, (3) reducing, and (4) categorizing data. The analysis was critically reviewed within the research group, which included members with diverse professional backgrounds in nursing and medicine, to ensure credibility. The data were paraphrased and coded using open coding techniques with the support of MAXQDA2020 software.

### 2.6 Ethical Considerations

All participants received written and oral information about the study and provided written consent after receiving detailed information about the study's purpose, procedures, voluntary nature, and their right to withdraw at any time without consequences. Participants were explicitly informed that they could refuse to answer any question or discontinue the interview at any point during data collection. No participants withdrew from the study.

In accordance with the Swiss Human Research Act, the responsible Ethics Committee issued a declaration of non‐responsibility (Req‐2024‐00234).

All audio recordings were deleted immediately after transcription. Transcripts are stored securely on password‐protected servers at Lucerne Cantonal Hospital, accessible only to the research team members directly involved in data analysis (M.Z.‐S. and L.V.). In accordance with institutional guidelines, data will be archived for 10 years and then permanently deleted.

## 3 Results

Data from 46 participants were included in the analysis (Table 1). Fourteen were patients, 17 were nurse anesthetists, and 15 were anesthesiologists. Participants varied in age and gender across the three groups. The interviews ranged in duration from 4 to 28 min.

**TABLE 1:** Characteristics of the study participants.

Profession	Patients	Nurse anesthetists	Anesthesiologists
Quantity (percentage)	14 (30.4%)	17 (36.9%)	15 (32.6%)
Women	4	12	6
Men	10	15	9
Age (years)			
Range	36–79	32–52	32–66
Median	62	39.5	46
Interview duration (minutes)			
Range	4–22	10–28	6–23
Median	8	14	15
Mean	11	15	16

Three main themes were identified for each group, yielding a total of nine themes. Subsequently, the findings will be presented by group: patients, then nurse anesthetists, and finally anesthesiologists.

### 3.1 Gaps in Care According to the Patients

The analysis of the patient interviews revealed three categories, namely: “information and preparation needs,” “pain,” and “recurring hospitalization” (Table 2).

**TABLE 2:** Categories and codes from patient interviews.

Category	Codes
Information and preparation needs	Feeling well looked after/being in good hands Not knowing what is coming up Being nervous/being anxious
Pain therapy	Treated during hospitalization Not prepared for treatment at home
Recurring hospitalization	Knowing yourself and your body Being familiar with processes Relatives lacking information Having to repeat and explain oneself

#### 3.1.1 Information and Preparation Needs

Most patients reported a positive experience with anesthetic care. Procedures were thoroughly explained during the pre‐anesthesia consultation. Patients felt well supported and reassured during surgery and in the recovery room afterward. Some patients experienced anxiety or fear before anesthesia or surgery. One patient undergoing surgery for carcinoma stated:

It is not just the anesthesia during which you are asleep. I find the “before” and “after” very important. (P.4)

Generally, patients did not really know what to expect before the anesthesia. They accepted the procedures and followed instructions—such as preoperative fasting—precisely. Some patients would have appreciated more advanced information about the time immediately after the operation and the remainder of that day. One patient, who had to undergo a cardiological procedure, felt unprepared and overwhelmed on the day of the operation:

How can I put it? I was taken a bit by surprise. I was in the middle of another appointment when it suddenly was my turn. Someone came into the room and said that I can now go to the operating theatre. I was unprepared. (P.7)

#### 3.1.2 Pain

About half of the participating patients explicitly mentioned pain and its management. During the hospital stay, pain was well treated. Analgesia was administered promptly in each case. The care provided by the pain nurses during a nerve catheter was appreciated. One patient, who had already undergone several operations, stated:

It went well so far. I did not have any extreme pain. Sometimes, it is a problem for me that I control myself too much. I made sure to speak up when it already hurt a little. I do not have to torture myself. (P.9)

Patients who had undergone orthopedic surgery reported that they received analgesics to take home. However, they were hardly instructed on how to take the medication for optimal pain management in everyday life. As a result, they experienced pain at home. One patient, who had a perioperative nerve catheter for pain management after surgery, described the following situation shortly before being discharged from the hospital:

In terms of the pain, it was very bearable, as I remember it now. However, when I got back home, the pain started, especially at night. Even now, I still have not completely gotten it under control. I feel like I am stuffed with medication. (P.13)

#### 3.1.3 Recurring Hospitalization

Patients with chronic conditions or multiple surgeries noticed differences in treatment. While first‐time patients were unfamiliar with hospital procedures, experienced patients had to repeat the same information, often without recognition of their prior medical history. One patient with multiple surgeries noted:

I had two operations within a month. The whole process started from scratch in the second operation. It was tedious. (P.4)

These patients knew their bodies well and considered themselves experts, particularly in positioning and medication management. They found it frustrating to repeat the same details. Communication gaps caused distress, as illustrated by a patient's wife who was left uninformed about a delayed surgery.

Most patients were asleep during the operation. Therefore, they considered anesthesia within the broader treatment experience, emphasizing the need for continuity of care, adequate pain management, including postoperative care, and recognition of their prior hospital experiences. They did not recall details of the anesthesia or its potential for optimization.

### 3.2 Gaps in Care According to the Experts

The experts, nurse anesthetists, and anesthesiologists surveyed identified gaps in care in the areas of “visibility of nursing,” “(interprofessional) practice development,” “pain therapy,” “changes in anesthesia,” “treatment process,” and “pain management” (Table 3).

**TABLE 3:** Categories and codes of interviews with experts.

Category	Codes
Nurse anesthetists	
Making nursing visible	Empowering patients, promoting self‐management (anxiety, postoperative nausea and vomiting, patient education) Recognizing needs for perioperative care, planning non‐pharmacological interventions Preoperative planning of postoperative care Patients with special needs (dementia, mental illness) need tailored care
(Interprofessional) practice development in the OR	Developing concepts interprofessional Involving families perioperatively Being aware of patients' exceptional situations/adapting communication Integration of anesthesia evaluation Increasing continuity in perioperative care (fragmented care)
Pain therapy	Treating patients with chronic pain in a person‐centered manner Pain therapy and care after removal of catheters
Anesthesiologists	
Changes in anesthesiology as a specialty	Increasingly elderly, multimorbid patients Prehabilitation Nobody feels responsible (fragmented care) Providing anesthesia on an outpatient basis More and more technically possible Fewer and fewer staff Underusing nursing skills Tying up resources through training Loss of knowledge through fluctuation
Anesthesiological treatment process	Only medical assessments Recognition of risks (frailty, chronic pain) Creation of perioperative treatment plans up to the ward Active management of the PACU Contact persons for patients outside office hours Fewer and fewer patients for whom standard procedures are sufficient Standardized postoperative ward rounds Patients trust nurses more than doctors
Pain therapy	Good standards as long as neural catheters in situ (standard care) Chronic pain therapy is undersupplied

#### 3.2.1 Nurse Anesthetists

##### 3.2.1.1 Making Nursing Visible

The category Making Nursing Visible highlights nurse anesthetists' perception that their specific contributions are consistently overlooked in anesthetic care. Two‐thirds of participants identified this as a problem. While core nursing values such as caring and social support are integral to anesthesia, they are overshadowed by technical tasks. The unique nursing role—such as patient‐centered communication, holistic care, and anxiety management—is not explicitly recognized, leading to a general lack of recognition for the anesthesia nursing profession. One participant explained:

What strikes me repeatedly in anesthesia is that often too little attention is paid to the human factor. Our professionals are very technically oriented. However, what if a patient is very anxious? It is about recognizing the human side of the patient. It is an area where we could do more. (N 6)

Many nurse anesthetists argued that anesthesia preparation is dominated by medical concerns, at the expense of patient empowerment and self‐management. Respondents called for greater focus on non‐pharmacological interventions to manage anxiety, pain, and postoperative nausea (e.g., aroma care, acupuncture, or suggestive communication). Some participants emphasized expanding anesthetic care to include the entire perioperative process—patient mobility and family support—especially for vulnerable groups like those with dementia or cognitive impairment. A nurse stated:

Preoperatively, specific patient populations receive too little patient education. Nursing topics such as coping with daily life are not covered. Patient education is not addressed preoperatively. This applies, for example, to patients with mental illnesses or dementia, or parents of disabled children. (N 5)

##### 3.2.1.2 Practice Development in the Operating Rooms (OR)

Participants noted that although hospital‐wide nursing guidelines for delirium and pressure ulcer prevention existed, they were rarely implemented in the anesthesia setting. Documentation of nursing interventions often ceased during anesthesia and only resumed after the patient was transferred back to the ward. One nurse anesthetist stated:

It is difficult to differentiate our (anesthesia) service from the other nursing care. Generally, anesthesia nursing is subsumed into the hospital‐wide relevant guideline. In addition, our role is often unclear. One example is the handover to the intensive care unit, where we communicate only a few nursing interventions. There is a gap here. Moreover, there is potential to become more involved in the future. It is important to document our contribution n to represent our performance clearly. (N 16)

Participants stressed that anesthesia constitutes an exceptional situation for patients, requiring sensitive and individualized communication. However, perioperative care guidelines were typically developed by physicians, with limited nursing input. Incorporating the nursing perspective added value to perioperative care. Consequently, preoperative visits were handled exclusively by physicians, whose focus remained on the anesthetic procedure rather than on patient concerns such as anxiety, mobility restrictions, or family involvement. Nurses noted a frequent lack of family integration during the perioperative phase. Structured follow‐up assessments could have improved practice. However, these interventions were often informal and lacked evaluation of care priorities. Nurses also identified the fragmentation of perioperative care as inefficiency. For example, separate staff members were often responsible for positioning, intraoperative care, and postoperative pain management, leading to communication gaps. A nurse anesthetist illustrates this:

The physicians drive many aspects. The patient's perspective is often neglected. One example in our clinic is the treatment of mental health patients, such as those who regularly require a gastroscopy. There is a lack of structured interdisciplinary collaboration and evidence‐based approaches to developing consolidated and clear standards for dealing with these patients. (N 12)

##### 3.2.1.3 Pain Therapy

Designated nurse anesthetists typically staffed Swiss pain management units within anesthesia departments. While about two‐thirds of respondents felt that care was adequate while nerve catheters were in place, they described post‐catheter care as unsystematic. Once catheters were removed, anesthesia personnel often no longer oversaw these patients. Participants noted that patients frequently desired follow‐up visits after catheter removal, as questions regarding pain self‐management often arose at this stage. Additionally, some nurse anesthetists felt that chronic pain management lacked a patient‐centered approach and sufficient preoperative planning.

Care by the pain nurses ends with the removal of catheters or pain pumps. At discharge, patients usually receive minimal information on continued pain management. This gap in the transition to aftercare means that many patients remain with untreated pain for too long, which increases the risk of chronic pain. (N 14)

#### 3.2.2 Anesthesiologists

##### 3.2.2.1 Changes in Anesthesiology as a Specialty

All interviewed anesthesiologists observed substantial shifts in the field. A central development is the growing shift toward outpatient surgery, which has altered the structure and continuity of care. One anesthesiologist from a regional hospital illustrates this dynamic:

As surgery is increasingly being performed on an outpatient basis, you must keep a close eye on how patients cope at home. You must be attentive when changing anything in the system. In outpatient surgery, the process does not end when the patient walks out. That is where there are gaps in care. (M 12)

Conversely, the inpatient surgery has become increasingly complex. Physicians explained that as standard cases move to outpatient settings, hospitalized patients are often severely ill and medically demanding. A physician from a university hospital points out:

In the inpatient area, we have a massive concentration of very sick people. Reasons for this include that every surgery that does not require an inpatient stay is performed in the outpatient setting. Thus, surgical interventions in inpatients are either large and stressful due to the procedure itself, or patients are already seriously ill beforehand. We no longer see the healthy ones. (M 7)

##### 3.2.2.2 Anesthesiological Treatment Process

Preoperative consultations were described as time‐limited and focused mainly on anesthesia risk assessment. Although strategies such as prehabilitation were known to improve outcomes, they were not widely implemented. Participants emphasized the importance of identifying broader perioperative risks, such as frailty, chronic pain, and nutritional status. One participant explained:

Another important point is determining a frailty score. It is important to consider the patient's condition and nutritional status at home. Although this goes beyond the scope of a normal anesthesia consultation, such an approach would fit well with the new focus on perioperative medicine. (M 10)

Continuity concerns were prominent. Several anesthesiologists noted that fear and uncertainty were sometimes left unaddressed due to time constraints or the lack of structured follow‐up. One physician described the risk of missing patient needs:

There are certainly one or two patients who need better treatment. Some patients are more afraid of the anesthetic than of the procedure itself. You can deal with this during the consultation, provided you have enough time and the necessary sensitivity. However, it is also possible that you determine the necessity of a second session, where you can do more. (M 1)

Staff shortages and high turnover rates among junior doctors complicated perioperative care. In contrast, the nursing workforce was seen as more stable. While the potential of nurses with advanced training was acknowledged, participants felt it was underutilized in practice. Specifically, two‐thirds of participants indicated that Postanesthesia Care Units (PACUs) were not used to their full potential. Transitions from the PACU to the ward were often poorly coordinated and lacked individualized treatment plans, leading to a loss of oversight. One anesthesiologist reflected:

The quality of postoperative care can be improved. In many places, the nursing staff in the recovery room care for patients on their own. As I have often experienced myself and heard from others, the recovery room is an unstable situation with little specialist knowledge. (…) Active management of the recovery room is often neglected. (…) Coordinating patients in the recovery room is an opportunity to improve care. The aim is to ensure that processes run smoothly: using scores to transfer patients promptly and to identify problems at an early stage. (M 4)

Furthermore, postanesthesia ward rounds were inconsistently implemented due to limited resources. Standardized feedback mechanisms were generally lacking:

I know that postanesthesia ward rounds are not carried out across the board in Switzerland. I also see gaps in care there. A clear feedback loop of what happens perioperatively is missing. (M 2)

##### 3.2.2.3 Pain Therapy

The importance of pain management was unanimously acknowledged. Where available, pain nurses monitored patients with regional anesthesia catheters. However, established pathways often did not account for complex cases or deviations from the norm. One pain management expert emphasized:

The (pain concept) works very well for standard procedures or procedures you know well. Standardization also applies to all aspects of regional anesthetics. Nevertheless, it sometimes gets difficult when there is a deviation from normal or standardized processes. (M 1, Pos. 11)

A specific concern was opioid weaning at discharge, which anesthesiologists felt was insufficiently addressed. Additionally, chronic pain care was described as fragmented, often involving long waiting times and minimal nursing involvement. Overall, respondents characterized the field as medically dominated, with significant room for improved interprofessional collaboration.

## 4 Discussion

This qualitative study explored perceived care gaps in anesthetic care from the perspectives of patients, nurse anesthetists, and anesthesiologists. We identified nine themes, three per stakeholder group, with pain management emerging as a universal concern. These findings are discussed in relation to the existing literature and their implications for integrating APNs into Swiss anesthetic care. Rather than discussing these themes sequentially, we synthesize our findings across stakeholder groups to reveal overarching patterns, critically analyze their significance in relation to the existing literature, and identify new knowledge gaps that warrant attention for both research and practice.

### 4.1 Pain Management as a Convergent Priority Across the Care Continuum

Perhaps the most striking finding is the convergence of pain management concerns across all three stakeholder groups, spanning the entire perioperative continuum. Patients reported inadequate preparation for home pain management despite good inpatient care. Nurse anesthetists noted unsystematic post‐catheter care, reflecting poor coordination between acute and chronic pain services. Anesthesiologists emphasized that established pathways fail to account for complex cases and opioid withdrawal. This multi‐stakeholder convergence is rarely documented in APN integration literature, which typically captures single perspectives (Van Hecke et al. 2023).

This convergence suggests that pain management gaps are systemic rather than isolated. The problem spans from preoperative planning through acute care to post‐discharge, aligning with international evidence of persistent inadequacies (Ke et al. 2023) but extending it by revealing recognition across the full continuum and all stakeholder groups. The distinct emphases reveal that “pain management” comprises multiple interrelated challenges. Walton et al. (2023) highlight that misconceptions about addiction and medication use hinder home management. Exactly the educational gap our patients identified. The Toronto Transitional Pain Service model (Katz et al. 2015) demonstrates that APNs can address all three levels simultaneously through patient education, systematic follow‐up protocols, and clinical expertise for complex cases. Recent findings confirm the effectiveness of APNs in coordinating perioperative pain management and opioid weaning within transitional pain services (Dunworth et al. 2024).

However, a critical gap remains: while APN pain coordination is documented in some contexts, evidence specific to the perioperative continuum in anesthesia services, particularly in European settings, is limited. How can systematic follow‐up be maintained when patients are discharged to various community settings? What infrastructure is needed to support APN coordination across institutional boundaries? These questions are particularly relevant for Switzerland, where formal transitional pain services do not yet exist. The convergence of stakeholder concerns makes pain management an ideal priority for initial APN integration efforts.

### 4.2 Systemic Fragmentation and the Challenge of Care Coordination

Our findings reveal fragmentation across multiple dimensions. Patients with recurring hospitalizations expressed frustration with having to provide the same information repeatedly and with a lack of continuity between encounters. Patients undergoing surgery felt unprepared for the postoperative period despite receiving procedural information. Anesthesiologists noted that time‐limited consultations focus narrowly on risk assessment, often overlooking broader concerns.

These findings converge on a systemic problem: anesthetic care is episodic and fragmented, lacking mechanisms for continuity of care. This mirrors Benham‐Hutchins et al.'s (2017) observation that patients with chronic diseases must “cobble together” their own records. Fragmentation is a critical safety risk in anesthesia, where unconscious patients cannot self‐advocate during handoffs. The information gaps align with those documented by Gobbo et al. (2020), who found inadequate preparation despite the availability of standard information. Our study extends this by revealing that consultations have evolved into complex encounters (medical, psychological, and social assessments) without corresponding increases in time (SSAPM 2025), thereby explaining the narrow focus on procedural risks. Bernstein et al. (2026) argue that preoperative care must be reshaped to address broader risks: cognitive dysfunction, nutritional status, and social vulnerability. The current lack of holistic assessment represents a failure to build patient resilience for surgery.

Fragmentation persists despite all stakeholders recognizing it as problematic, suggesting structural rather than individual problems. International evidence shows APNs can provide continuity across fragmented episodes (Assolari et al. 2024; Lekens et al. 2023). However, a critical gap concerns how to implement APN coordination in inherently episodic care. Our findings suggest that APNs could conduct comprehensive preoperative assessments that extend beyond risk evaluation (Bernstein et al. 2026), serve as “continuity anchors” for recurring patients with longitudinal records, and provide transitional care follow‐up. Implementation requires not only creating positions, but also developing infrastructure to support coordination across episodic encounters.

### 4.3 Nursing Contributions, Visibility, and Practice Development

Nurse anesthetists identified that their contributions, such as patient‐centered communication, anxiety management, and holistic care, were often overlooked. They also found that nursing guidelines (e.g., for delirium or pressure ulcer prevention) were rarely implemented, and that there was minimal nursing input in the development of perioperative guidelines. These findings are interconnected: the invisibility of nursing contributions translates into structural exclusion from practice development, thereby perpetuating the undervaluation of nursing expertise.

This resonates with Wising et al. (2024), who documented how power dynamics render nursing contributions invisible, with caring work undervalued compared to technical tasks. Olin et al. (2022) documented that Swedish nurse anesthetists perform 98 tasks per hour during induction, leaving no capacity for practice development despite possessing relevant expertise. This paradox, nurses with specialized knowledge excluded from processes that could benefit from their expertise, has tangible consequences. Nurse anesthetists identified specific structural deficits in the Swiss PACU context, where patients are rarely extubated, and the department functions as a temporary waiting area with few standardized examinations, resulting in significant disruption of care continuity.

The invisibility problem raises a critical question: How can we ensure APN contributions are made visible from the outset, rather than reproducing the same patterns? APN integration should explicitly include strategies to make nursing contributions visible through documentation and quality metrics (Kleinpell et al. 2019). APNs could lead evidence‐based projects in areas such as the PACU to ensure nursing guidelines are maintained perioperatively. However, would creating APN roles provide infrastructure for practice development, or would APNs face the same constraints? International experience suggests that explicit inclusion of practice development in role descriptions, with protected time, is essential (Tracy et al. 2022). Without structural support, APNs may become additional clinical staff rather than practice development leaders.

### 4.4 Evolving Specialty Demands and New Care Models

Anesthesiologists noted significant changes, particularly the shift toward outpatient surgery and concentration of multimorbid patients in inpatient facilities. Vetter et al. (2024) argue that the future of outpatient anesthesia requires entirely new care models that extend beyond facilities to monitor home recovery. APNs could provide pre‐admission assessments, patient education for home recovery, and post‐discharge follow‐up. Given that approximately 20% of palliative hospitalizations in Switzerland are potentially avoidable and many patients prefer home‐based care (Hurni et al. 2024), integrating APNs into outpatient palliative teams could improve care quality and continuity. Nevertheless, research into the exact implementation of APN roles to prevent such avoidable admissions is still required as the shift toward outpatient care accelerates.

### 4.5 Implications for APN Integration in the Swiss Anesthesia Context

The identified gaps present specific opportunities for targeted APN integration. Building on advanced clinical competencies (Schober et al. 2020), APNs could bridge critical service gaps through roles spanning the perioperative continuum. Preoperatively, this involves conducting holistic visits tailored to patient needs (Bernstein et al. 2026). In the operating room, APNs can lead practice development projects to ensure the systematic implementation of evidence‐based nursing guidelines, provided they have protected time for these activities. Postoperatively, they address PACU structural deficits, provide systematic pain follow‐up (Katz et al. 2015; Dunworth et al. 2024), and act as “continuity coordinators” for recurring patients to ensure seamless transitions in care.

The Swiss context appears favorable, as anesthesiologists showed openness to APN roles, contrasting with resistance documented elsewhere (Weinberg et al. 2020). However, successful implementation requires clear role definitions to avoid ambiguity (Kleinpell et al. 2019). Evidence suggests that full integration can require 15–20 years, necessitating tripartite approaches between managers, practitioners, and educators (Unsworth et al. 2024). Currently, Switzerland lacks the regulatory frameworks, specific educational programs, and reimbursement structures for anesthesia APNs, presenting both challenges and opportunities for future‐oriented healthcare policy.

### 4.6 Contribution to Knowledge

This study makes several novel contributions. First, the multi‐stakeholder convergence on pain management as a priority is rarely documented, suggesting this as a critical focus for APN roles. Second, we extend care fragmentation concepts to the episodic context of anesthesia, identifying APNs as “continuity anchors.” Third, we document Swiss anesthesiologists' openness to APN integration, suggesting favorable cultural conditions for the introduction of the role. Fourth, we identify specific operational gaps rather than generic descriptions, providing a targeted roadmap for clinical implementation. Finally, we contribute to the limited literature on APN integration in Swiss healthcare, providing essential baseline data before formal roles are introduced.

### 4.7 Trustworthiness and Limitations

This study draws on the perspectives of patients, nurse anesthetists, and anesthesiologists and provides a comprehensive overview of gaps in care. Recruitment via professional networks ensured geographical and institutional diversity. Nevertheless, the sample size was limited, and the shorter interview duration may have limited the depth of the findings. In particular, one patient provided an interview of only 4 min, which represents a notable limitation of our data collection. The relatively short interview duration overall reflects the focused nature of the interview guides, which concentrated on specific aspects of experiences with anesthetic care and perceived gaps rather than general life stories. Despite the shorter duration, the interviews provided rich, focused data on the research questions, though the 4‐min interview may have constrained the depth of insights from that participant. The inclusion of the patient perspective is a notable strength, but future studies should examine whether the findings also apply to different levels of health literacy. As this study surveyed patients at only one location in German‐speaking Switzerland, its transferability is limited. To address this, we used a targeted sample selection that maximized variation and provided detailed descriptions of the context and participants, enabling readers to assess applicability. The consistency of our results with the international literature (see Discussion) underscores their broader relevance.

Transparency in data analysis was ensured through systematic coding and peer discussion. Nevertheless, as a qualitative descriptive study, causal relationships and interactional dynamics could not be deeply examined.

## 5 Conclusions

Anesthesia has evolved into perioperative medicine. Further changes in contextual factors require adaptations in the services provided by anesthesia, including ambulatory care. A new model of care integrating the APN anesthesia offers a potential solution to address these gaps. This study highlights the areas in which APN anesthesia can be used, with its nursing focus: preoperative patient preparation, including prehabilitation; patient education for postoperative care; the PACU; and postoperative or palliative pain management. The provision of anesthetic care by APNs also contributes to the development of intraoperative interprofessional practice. An APN anesthesia can enhance patient‐centered care when integrated across sectors into outpatient care.

### 5.1 Relevance for Clinical Practice

The findings of this study show the need to adapt anesthetic care to evolving clinical, demographic, and systemic challenges. Patients, nurse anesthetists, and anesthesiologists identified gaps, highlighting missed opportunities in patient preparation, interprofessional practice development, clinical leadership in Postanesthesia Care Units, and pain management. Integrating APNs into anesthetic care offers a promising way to address these deficits. APNs can enhance patient‐centered preoperative care, especially for people with chronic conditions. They can also lead to improvements in postoperative and palliative pain management. Their involvement may strengthen interprofessional collaboration, improve care continuity, and help make anesthetic care more responsive and future‐ready. To implement this effectively, clinical settings must address role clarity, team integration, and develop supportive policy frameworks.

## Author Contributions

**Maya Zumstein‐Shaha:** conceptualization, writing – review and editing, formal analysis, supervision, validation, methodology. **Luzia Vetter:** conceptualization, writing – original draft, writing – review and editing, formal analysis, investigation, project administration, visualization, methodology.

## Funding

The authors have nothing to report.

## Ethics Statement

In accordance with the Swiss Human Research Act, the Ethics Committee of Northwestern and Central Switzerland issued a declaration of non‐responsibility (Req‐2024‐00234).

## Consent

Informed consent was obtained from all individual participants included in the study. Participants were informed about the aim, procedure, voluntary nature, and data handling of the research.

## Conflicts of Interest

The authors declare no conflicts of interest.

## Supporting information


**Data S1:** nhs70317‐sup‐0001‐Supinfo.docx.

## Data Availability

All data generated and analyzed during this study are included in this published article. Due to the qualitative nature of the data (interview transcripts) and to protect participant confidentiality, raw data are not publicly available. Access to anonymized excerpts may be granted upon reasonable request to the corresponding author.
