# Supplementary material for: Integrating Advanced Practice Nurses in Anesthesia to Tackle Gaps in Current Health Care: A Qualitative Study
Source: Nurs Health Sci. 2026 Mar 8;28(1):e70317. doi: 10.1111/nhs.70317 (PMC12968485; doi:10.1111/nhs.70317)
Supplement: Supplementary file 1 — Data S1: nhs70317‐sup‐0001‐Supinfo.docx. [file NHS-28-e70317-s001.docx]

Supplementum

Excerpt from the interview guide

| Expert survey (Anesthesiologists, Nurse Anesthetists) |
| --- |
| Where do you see gaps or potential for optimization in current patient care in anesthesia?  Where do you think patients still have unmet needs in terms of anesthesia care? |
| Patients |
| How did you experience the care provided by the anesthesia department, starting with the pre-operation briefing to the recovery room and pain management?  What do you think anesthesia should do even better? What needs should it address even more? |
